# Ultrasound-Guided Femoral Hemostasis in Peripheral Angioplasty: Real-World Outcomes with Vascular Closure Devices Versus Manual Compression

**DOI:** 10.3390/medicina62010028

**Published:** 2025-12-23

**Authors:** Ioannis Skalidis, Livio D’Angelo, Mariama Akodad, Youcef Lounes, Hakim Benamer, Benjamin Honton, Antoine Sauguet, Neila Sayah, Pietro Laforgia, Nicolas Amabile, Thomas Hovasse, Philippe Garot, Antoinette Neylon, Francesca Sanguineti, Stephane Champagne, Thierry Unterseeh

**Affiliations:** 1 Department of Cardiology, HFR Fribourg—Cantonal Hospital, University of Fribourg, 1700 Fribourg, Switzerland; 2 Institut Cardiovasculaire Paris-Sud, Hôpital Jacques Cartier, 91300 Massy, France; 3 Department of Cardiology, Clinique Pasteur, 31076 Toulouse, France; bhonton@clinique-pasteur.com (B.H.);

**Keywords:** vascular closure device, femoral access, peripheral artery disease, peripheral intervention, restenosis, complications

## Abstract

*Background and Objectives:* Access-site complications (ASCs) remain clinically relevant after peripheral endovascular procedures, particularly with large femoral sheaths and complex anatomy. While randomized coronary trials show non-inferiority of vascular closure devices (VCDs) versus manual compression (MC), real-world data in peripheral interventions performed under systematic ultrasound-guided access are limited. *Materials and Methods*: This retrospective single-center cohort included consecutive peripheral arterial revascularizations (2010–2023) performed via common femoral access under real-time ultrasound guidance. Hemostasis was achieved using MC or VCDs, categorized as collagen plug-based, suture-mediated, or clip-based systems. The primary endpoint was 30-day ASCs, defined as hematoma requiring management, pseudoaneurysm, bleeding requiring transfusion, access-site thrombosis/occlusion, arteriovenous fistula, or infection. The secondary endpoint was VCD failure, defined as unsuccessful hemostasis requiring adjunctive measures. Multivariable logistic regression adjusted for prespecified anatomical and procedural covariates, including sheath size > 6 Fr and puncture-site calcification. *Results*: Among 231 procedures, VCDs were used in 139 (60.2%) and MC in 92 (39.8%). ASC occurred in 28 cases (12.1%), with higher rates in the MC group compared with VCDs (18.5% vs. 9–14% across device types; *p* = 0.044). In adjusted analyses, MC (vs any VCD) (odds ratio [OR] 2.41, 95% confidence interval [CI] 1.06–5.47; *p* = 0.035), sheath size > 6 Fr, and puncture-site calcification were independently associated with ASCs. VCD failure occurred in 5 cases (3.6%) and was not observed with collagen plug-based devices. *Conclusions*: In this ultrasound-guided real-world peripheral cohort, VCD use was associated with lower 30-day ASC rates and low device failure rates compared with MC. Given the retrospective and non-randomized design, these findings should be considered hypothesis-generating and support individualized, imaging-guided strategies for femoral closure in peripheral interventions.

## 1. Introduction

Vascular closure devices (VCDs) are widely used to achieve hemostasis following percutaneous vascular procedures and have progressively supplanted manual compression in many interventional fields. Their increasing adoption is driven by several practical advantages, including reduced time to ambulation, improved patient comfort, and decreased demands on nursing resources [1,2]. In the coronary setting, multiple randomized trials and meta-analyses have demonstrated that VCDs are generally safe and effective, with complication rates comparable to or lower than those observed with manual compression.

In peripheral arterial interventions, however, the evidence base is considerably less robust. Access-site complications (ASCs)—including hematomas, pseudoaneurysms, arteriovenous fistulas, bleeding requiring transfusion, and femoral artery thrombosis—remain clinically relevant events that may prolong hospitalization, increase healthcare utilization, and adversely affect limb function [3,4]. Reported ASC rates range from 1% to 6%, with risk strongly influenced by sheath diameter, puncture location, the presence of arterial calcification, and comorbidities such as diabetes and chronic kidney disease [5,6]. Compared with coronary procedures, peripheral interventions frequently require larger introducer sheaths, longer procedure durations, and treatment of heavily calcified arterial segments, all of which may increase vulnerability to femoral access complications. Furthermore, the anatomical and procedural complexity of lower-limb arterial disease—including multilevel disease patterns and extensive calcification—has been well documented in seminal trials such as the superficial femoral artery stent studies by Schillinger et al. [7], underscoring the fundamentally different vascular environment in which closure devices must operate.

Despite the widespread use of VCDs, comparative data on device performance in peripheral interventions remain limited and heterogeneous. Direct comparisons between collagen plug-based, suture-mediated, and clip-based systems are sparse, and existing studies suggest that each device category may have distinct failure mechanisms, particularly in anatomically complex femoral punctures, calcified common femoral arteries, or antegrade access trajectories. In addition, delayed complications such as device failure, infection, access-site occlusion, or pseudoaneurysm formation have been reported, highlighting the need for device selection tailored to arterial morphology and puncture characteristics [5,6].

A major limitation of prior evidence is the inconsistent use of real-time ultrasound guidance for femoral access. Ultrasound-guided puncture improves identification of the common femoral artery, reduces inadvertent high or low access, and may lower the risk of access-site complications. However, many previous peripheral cohorts combine ultrasound-guided and landmark-based access, limiting interpretation of closure device performance under optimized puncture conditions. Real-world data from centers employing systematic ultrasound guidance for all femoral accesses remain particularly scarce.

Furthermore, very few studies have assessed closure device performance longitudinally in a single contemporary peripheral arterial cohort with uniform imaging-guided access techniques. Peripheral interventions differ fundamentally from coronary procedures in sheath size, calcification severity, biomechanical forces, and clinical complexity; therefore, extrapolation from coronary VCD trials to peripheral revascularization is uncertain. These gaps underscore the need for contemporary, pragmatic analyses to inform device selection, risk stratification, and access-site management in peripheral vascular interventions.

In this context, we investigated short-term femoral access-site safety in a real-world cohort of peripheral arterial endovascular revascularizations performed with systematic ultrasound-guided common femoral artery puncture. This study provides comparative data across three VCD categories—collagen plug-based, suture-mediated, and clip-based—alongside manual compression. By focusing on a large, longitudinal, real-world population treated under standardized imaging-guided access conditions, the analysis aims to clarify the performance of different closure strategies and identify predictors of access-site complications relevant to contemporary practice.

## 2. Materials and Methods

### 2.1. Study Design and Setting

This retrospective, single-center observational cohort study was conducted at the Institut Cardiovasculaire Paris Sud (ICPS), Hôpital Privé Claude Galien, Quincy-sous-Sénart, France. All consecutive peripheral arterial endovascular procedures requiring common femoral artery (CFA) access between January 2010 and October 2023 were identified from a prospectively maintained institutional registry. This registry systematically captures demographic characteristics, comorbidities, imaging findings, procedural details, and early post-procedural outcomes. The study complied with institutional and national ethical standards, was approved by the local ethics committee, and all patients provided written informed consent authorizing the use of anonymized data for research purposes.

### 2.2. Study Population

Eligible participants were adults aged ≥18 years who underwent peripheral endovascular revascularization for symptomatic lower-limb ischemia caused by ≥50% stenosis, confirmed by duplex ultrasound, invasive angiography, or computed tomography angiography. Procedures were included only when CFA access and a clearly documented hemostasis method were present. To ensure procedural homogeneity, patients treated via radial, brachial, or popliteal access were excluded, as were patients undergoing surgical (non-endovascular) revascularization or hybrid procedures. At our institution, alternative access routes are frequently used in peripheral endovascular practice. In institutional data from the same center and overlapping study period, upper-limb access (radial or brachial) accounted for approximately 28.5% of first-attempt access sites. Accordingly, the present analysis represents a selected cohort of procedures performed via common femoral artery access. Of 285 peripheral endovascular procedures initially identified, 54 were excluded based on predefined criteria, including non-common femoral artery access (radial, brachial, or popliteal), surgical or hybrid procedures, and incomplete procedural documentation or missing 30-day follow-up. The final analytic cohort therefore comprised 231 consecutive procedures performed via common femoral artery access with complete 30-day follow-up. Post-procedural follow-up was performed in accordance with institutional practice, generally combining in-hospital clinical assessment, telephone contact, and outpatient review.

### 2.3. Ultrasound-Guided Femoral Access

All procedures employed real-time ultrasound guidance for arterial puncture, following a standardized institutional technique throughout the study period. High-frequency linear transducers (7–12 MHz) were used to visualize the CFA in both transverse and longitudinal planes. Operators routinely identified the CFA bifurcation, the inguinal ligament projection, arterial depth, the position of the femoral vein, and the presence and distribution of anterior or posterior wall calcification. Puncture-site calcification was assessed primarily by real-time ultrasound and recorded as a binary variable (present/absent) based on the presence of echogenic plaque with acoustic shadowing at the intended puncture site; fluoroscopy and pre-procedural computed tomography angiography, when available, were used as complementary sources of information. The arterial puncture was performed at the optimal segment between the inguinal ligament and the bifurcation to reduce the risk of high or low puncture. When calcification was present, particular attention was paid to avoiding heavily calcified segments and posterior plaque. A micropuncture system was commonly used to minimize arterial trauma, and needle entry was performed under direct ultrasound visualization. Wire advancement and sheath placement were confirmed by ultrasound or fluoroscopy.

### 2.4. Vascular Access and Hemostasis Technique

All interventions were performed by experienced interventional operators with several years of independent practice. CFA access was obtained using either a retrograde or antegrade approach depending on anatomical requirements. Intravenous unfractionated heparin was administered according to lesion complexity, with activated clotting time measured when appropriate to guide sheath removal. Activated clotting time values at sheath removal were not systematically recorded across the study period and were therefore not available for uniform quantitative analysis.

Hemostasis was achieved using either manual compression (MC) or one of three categories of vascular closure devices (VCDs). Manual compression was applied immediately after sheath removal, typically for a minimum of 15 min, followed by immobilization and clinical monitoring. When a VCD was selected, device choice reflected real-world practice and operator judgment, taking into account vessel size, sheath diameter, access depth, access-site calcification, and puncture trajectory. Collagen plug-based devices (e.g., Angio-Seal) function through a bioabsorbable intraluminal anchor coupled with an extravascular collagen plug. Suture-mediated devices (e.g., Perclose ProGlide) close the arterial puncture mechanically through one or more sutures and were used with a pre-closing strategy for larger sheaths when indicated. Clip-based systems (e.g., StarClose) achieve hemostasis through an externally deployed nitinol clip designed to approximate the arterial wall without entering the lumen. All devices were deployed according to manufacturer instructions and institutional standards.

### 2.5. Outcome Measures

The primary outcome was any access-site complication (ASC) occurring within 30 days of the index procedure. Complications included hematomas requiring management—defined as hematomas prompting additional or prolonged manual compression beyond routine post-procedural care, unplanned clinical evaluation or imaging, or escalation of care including transfusion or surgical/endovascular intervention—pseudoaneurysms confirmed clinically or by duplex ultrasonography, bleeding events requiring transfusion, access-site arterial thrombosis or occlusion causing ischemic symptoms, arteriovenous fistulas, and local infections requiring antibiotic therapy or drainage. The secondary outcome was VCD failure, defined as the inability to achieve immediate hemostasis, necessitating adjunctive manual compression, a second closure device, or surgical or endovascular salvage. When in-stent restenosis was present, it was recorded only when affecting the target vessel undergoing treatment.

### 2.6. Follow-Up and Post-Procedural Assessment

All patients underwent structured clinical assessment immediately after the procedure, during hospitalization, and again within 30 days. Follow-up evaluations included inspection of the access site, palpation of distal pulses, assessment for groin pain or swelling, and measurement of the ankle–brachial index when indicated. Duplex ultrasound was performed selectively in the presence of pulsatile masses, persistent pain, signs of limb ischemia, or suspected pseudoaneurysm. Imaging was not used routinely in asymptomatic patients, reflecting standard clinical practice.

### 2.7. Data Management and Statistical Analysis

Clinical, anatomical, procedural, and outcome data were extracted from the registry and cross-checked against procedure reports, electronic medical records, nursing notes, and discharge summaries. Two investigators independently validated data accuracy and consistency. Continuous variables were expressed as the mean ± standard deviation or median with interquartile range, depending on normality assessed by the Shapiro–Wilk test. Categorical variables were summarized as absolute numbers and percentages. Between-group comparisons were performed using the *t*-test or Mann–Whitney U test for continuous variables and χ^2^ or Fisher’s exact test for categorical variables. Multivariable logistic regression was performed to identify independent predictors of 30-day ASCs. Covariates were selected a priori based on biological plausibility and prior literature and included age, sex, body mass index, access side, antegrade versus retrograde approach, sheath size > 6 Fr, access-site calcification, and access vessel diameter. Missing data were minimal; therefore, complete-case analysis was used. A two-sided *p*-value < 0.05 was considered statistically significant. All statistical analyses were performed using SAS software version 9.4 (SAS Institute, Cary, NC, USA).

## 3. Results

### 3.1. Study Population

**Figure 1:**
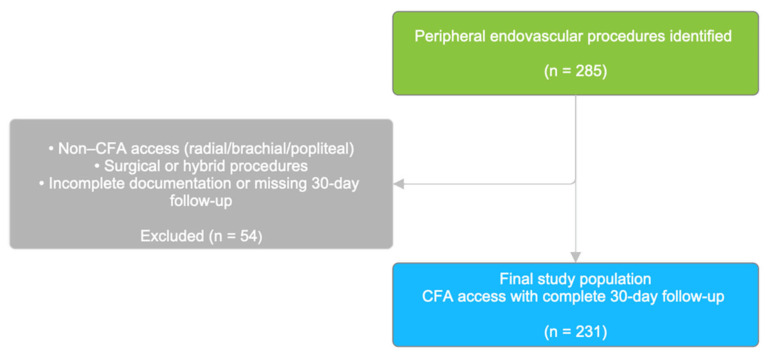
Study Flowchart.

A total of 231 patients underwent peripheral endovascular revascularization via common femoral artery access between January 2010 and October 2023 and were included in the analysis. Patient selection and exclusions are summarized in Figure 1.

The mean age was 71.4 ± 10.1 years, and 139 patients (60.2%) were male. Manual compression was used in 92 procedures (39.8%), whereas vascular closure devices (VCDs) were used in 139 procedures (60.2%). Among the VCD subgroup, 89 patients (64.0%) received a collagen plug-based device, 30 (21.6%) were treated with a suture-mediated system, and 20 (14.4%) with a clip-based device.

Baseline demographic, clinical, anatomical, and procedural characteristics are summarized in Table 1. Age, sex distribution, cardiovascular risk factors, and comorbidity profiles were similar across the four groups. Lesion characteristics, including the presence of chronic total occlusion and in-stent restenosis, showed comparable distributions. Access vessel diameter and lesion length were likewise similar among groups. Sheath size > 6 Fr was present in 46.7% of MC cases, 40.4% of collagen plug cases, 43.3% of suture-mediated cases, and 45.0% of clip-based cases. Calcification at the puncture site was documented in 37.0%, 32.6%, 36.7%, and 40.0% of cases, respectively.

### 3.2. Access-Site Complications

Access-site complications (ASCs) occurred in 28 of 231 procedures (12.1%) within 30 days. Hematomas requiring medical management were observed in 13 cases (5.6%), pseudoaneurysms in 8 cases (3.5%), and access-site thrombosis or occlusion in 4 cases (1.7%). No access-site infections or arteriovenous fistulas were recorded.

**Figure 2:**
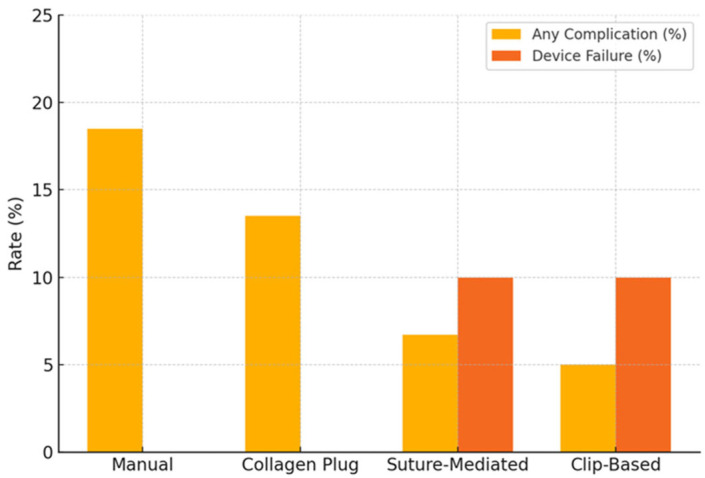
Early Access-Site Complications and Device Failure by Closure Strategy. A bar chart displaying the incidence of early access-site complications (within 30 days) and device failure stratified by closure method. Manual compression was associated with the highest overall complication rate, while collagen plug-based devices demonstrated the lowest complication and failure rates. Device failure occurred exclusively with suture- and clip-based systems.

ASC rates according to hemostasis strategy were as follows: 18.5% in the manual compression group (17 of 92), 13.5% in the collagen plug group (12 of 89), 6.7% in the suture-mediated group (2 of 30), and 5.0% in the clip-based group (1 of 20). Detailed distributions of complication types by closure strategy are provided in Table 2 and Figure 2.

### 3.3. Closure Device Failure

Among the 139 procedures in which a VCD was used, closure device failure occurred in 5 cases (3.6%). Failure occurred in 3 of 30 suture-mediated devices (10.0%) and in 2 of 20 clip-based devices (10.0%). No failures were recorded among the 89 collagen plug-based devices (0%). Device failure rates for all device categories are reported in Table 3. Comparisons among individual vascular closure device categories are limited by small subgroup sizes and should therefore be interpreted as exploratory and hypothesis-generating.

### 3.4. Predictors of Access-Site Complications

Multivariable logistic regression analysis identified three variables independently associated with 30-day ASCs (Table 3). Manual compression, relative to any VCD, was associated with an odds ratio (OR) of 2.41 (95% confidence interval [CI] 1.06–5.47; *p* = 0.035). Sheath size > 6 Fr was associated with an OR of 2.12 (95% CI 1.01–4.47; *p* = 0.048). Calcification at the puncture site was associated with an OR of 2.74 (95% CI 1.29–5.81; *p* = 0.009).

Access vessel diameter, chronic total occlusion, and in-stent restenosis were not significantly associated with ASCs in the adjusted model.

## 4. Discussion

In this contemporary real-world cohort of 231 peripheral endovascular procedures performed with systematic ultrasound-guided common femoral artery (CFA) access, vascular closure device (VCD) use was associated with lower observed 30-day access-site complication (ASC) rates compared with manual compression. Overall, 12.1% of procedures were complicated by an access-site event, with complication rates ranging from 18.5% in the manual compression group to 5.0–13.5% across VCD categories. These findings align with contemporary peripheral endovascular studies reporting low overall rates of femoral access complications when closure strategies are applied selectively and under optimized access conditions [3,4,5,6,8].

The primary endpoint was assessed using a composite ASC definition incorporating hematoma requiring management, pseudoaneurysm, bleeding requiring transfusion, access-site thrombosis or occlusion, arteriovenous fistula, and infection. Composite femoral access-site endpoints are commonly used in peripheral endovascular registries and observational cohorts to enable comparative analyses where individual events are infrequent [3,4,5,6,8]. Prior peripheral studies evaluating femoral access safety after lower-extremity revascularization have similarly relied on composite outcomes encompassing hematoma and vascular injury, while acknowledging heterogeneity in event definitions and follow-up strategies [4,5,6]. Importantly, in the present study, all individual components of the composite endpoint were reported separately, allowing for assessment of their respective clinical relevance.

Beyond hemostasis strategy, anatomical and procedural factors emerged as key determinants of ASC risk. After multivariable adjustment, sheath size > 6 Fr and puncture-site calcification were independently associated with higher complication rates. These findings are consistent with prior peripheral registry data identifying larger sheath diameter, severe calcification, and complex access anatomy as major contributors to femoral access complications following peripheral vascular intervention (PVI) [3,5,8]. In peripheral practice—where larger sheath sizes are frequently required for complex revascularization—these factors likely reflect increased arteriotomy complexity and impaired arterial compliance, underscoring the need for anatomy-tailored access and closure strategies.

Manual compression, compared with any VCD, remained associated with a more than twofold increase in ASC risk after adjustment for key confounders. While causality cannot be inferred in this non-randomized observational study, similar associations have been reported in peripheral cohorts, where manual compression is often preferentially selected in anatomically unfavorable access sites, including deep vessels, heavy calcification, or suboptimal puncture geometry [4,5]. Accordingly, the higher complication rates observed with manual compression may partly reflect residual confounding by indication rather than intrinsic inferiority of the technique, reinforcing the importance of individualized hemostasis selection.

Differences among VCD subgroups were also observed. Collagen plug-based systems demonstrated no device failures in this cohort, whereas failure occurred in 10% of both suture-mediated and clip-based systems. Although this study was not powered for direct device-level comparisons, these numerical patterns are biologically plausible and consistent with known differences in closure mechanisms. Collagen plug-based devices rely on an intraluminal anchor and extravascular collagen seal, which may be less sensitive to minor variations in tract alignment or puncture depth. In contrast, suture- and clip-based systems require precise tissue capture and may be more susceptible to failure in the presence of calcification or unfavorable access geometry [6,8]. The absence of pseudoaneurysm or thrombosis events in the suture-mediated and clip-based groups suggests that most failures were mechanical rather than ischemic or structural in nature; however, given the limited subgroup sizes, these findings should be interpreted as exploratory.

Systematic ultrasound guidance represents a major strength of the present study. Ultrasound-guided femoral access improves visualization of the CFA bifurcation, arterial depth, venous overlap, and calcification distribution, thereby reducing high or low puncture and inadvertent venous injury. Randomized and observational studies have demonstrated improved access accuracy and reduced vascular complications with ultrasound guidance, including in peripheral and mixed procedural cohorts [9,10,11]. In peripheral-specific series, routine ultrasound guidance has been associated with lower rates of groin hematoma following PVI [10]. The relatively low rates of pseudoaneurysm and access-site thrombosis observed in the present cohort may therefore reflect consistent application of a standardized ultrasound-guided access protocol.

From a clinical perspective, these findings support a pragmatic approach to femoral hemostasis in contemporary peripheral interventions. In an era characterized by increasing procedural complexity, larger sheath requirements, and older, frailer patient populations, VCDs—when selected appropriately and deployed under ultrasound guidance—appear to provide effective hemostasis with acceptable safety. Integration of structured access protocols, routine ultrasound guidance, and anatomy-based device selection may further optimize access-site outcomes across peripheral endovascular programs.

Future investigations should focus on prospective studies comparing closure strategies across well-defined anatomical phenotypes, including severe CFA calcification, obesity with deep access tracts, and antegrade CFA puncture, where complication risk is traditionally higher. Longer-term follow-up incorporating systematic duplex ultrasound evaluation may further clarify the incidence of late access-site sequelae, such as delayed pseudoaneurysm formation or arterial stenosis, and refine the role of closure devices in peripheral vascular practice.

### Limitations

This study has several important limitations that merit consideration. First, the retrospective, single-center observational design inherently carries risks of unmeasured confounding and selection bias. The choice between manual compression and vascular closure device (VCD) use was not randomized and reflected operator judgment based on anatomical and procedural factors. In routine practice, manual compression was more frequently selected in patients with challenging access characteristics, such as deeper vessels, heavier calcification, or less favorable puncture geometry. Although puncture-site calcification and other procedural variables were included in multivariable analyses, residual confounding related to anatomical complexity or operator preference cannot be fully excluded and may have influenced the observed differences in complication rates. Second, while the overall sample size was moderate, the number of patients treated with individual VCD categories—particularly suture-mediated and clip-based devices—was relatively small. This limited statistical power to detect uncommon adverse events and precluded definitive device-level comparisons. Accordingly, findings related to differences among VCD subtypes should be interpreted cautiously and considered hypothesis-generating rather than conclusive. Third, the long enrollment period spanning 13 years introduces potential heterogeneity related to evolving operator experience, procedural techniques, peri-procedural management, and successive generations of vascular closure devices. Although this temporal variability may limit direct extrapolation to current-generation devices, it also reflects real-world practice across different eras and enhances the external validity of the findings. Nevertheless, outcomes observed in earlier phases of the study period may not fully represent contemporary device performance. Fourth, although all femoral punctures were performed under ultrasound guidance, the study could not account for potential variability in ultrasound technique, operator proficiency, puncture depth, or angle of entry, factors that may influence access-site outcomes. Additionally, procedural variables such as sheath dwell time or subtle differences in puncture location relative to the CFA bifurcation were not systematically captured. Fifth, follow-up was limited to 30 days, and the study did not assess late access-site events, including delayed pseudoaneurysm formation, late arterial stenosis, aneurysmal remodeling, or access-related neuropathic complications. As such, the analysis primarily reflects early access-site safety rather than long-term vascular sequelae. Moreover, periprocedural antithrombotic management—including antiplatelet therapy, oral anticoagulation, bridging strategies, and activated clotting time at sheath removal—was not protocolized and was not systematically recorded in the registry across the entire study period. This limited the ability to adjust for these important bleeding-related confounders, and residual confounding related to variability in anticoagulation strategies cannot be excluded. Finally, post-procedural imaging follow-up was not performed routinely and was instead guided by clinical indication. As a result, asymptomatic or subclinical access-site complications, such as small pseudoaneurysms not associated with symptoms, may have been underdiagnosed, introducing the potential for ascertainment bias.

## 5. Conclusions

In this real-world cohort of peripheral endovascular interventions performed with systematic ultrasound-guided femoral access, vascular closure device use was associated with lower observed 30-day access-site complication rates compared with manual compression. Given the retrospective and non-randomized design, these findings should be considered hypothesis-generating and support an individualized, anatomy-guided approach to femoral hemostasis rather than definitive practice recommendations. Prospective, adequately powered studies are needed to define optimal closure approaches in contemporary peripheral vascular interventions.

## Figures and Tables

**Table 1:** Baseline Clinical, Anatomical, and Procedural Characteristics.

Variable	Manual Compression (*n* = 92)	Collagen Plug VCD (*n* = 89)	Suture-Mediated VCD (*n* = 30)	Clip-Based VCD (*n* = 20)	*p*-Value
Number of patients	92	89	30	20	
Age (years), mean ± SD	72.1 ± 9.8	70.5 ± 10.4	71.0 ± 10.9	71.6 ± 9.4	0.48
Male sex, *n* (%)	55 (59.8%)	55 (61.8%)	18 (60.0%)	11 (55.0%)	0.79
BMI (kg/m^2^), mean ± SD	27.2 ± 4.6	26.5 ± 4.1	26.7 ± 4.3	26.9 ± 4.5	0.36
Hypertension, *n* (%)	66 (71.7%)	63 (70.8%)	22 (73.3%)	15 (75.0%)	0.94
Diabetes mellitus, *n* (%)	29 (31.5%)	24 (27.0%)	10 (33.3%)	6 (30.0%)	0.73
Dyslipidemia, *n* (%)	59 (64.1%)	56 (62.9%)	20 (66.7%)	13 (65.0%)	0.81
Current smoker, *n* (%)	39 (42.4%)	40 (44.9%)	14 (46.7%)	10 (50.0%)	0.91
Chronic kidney disease, *n* (%)	27 (29.3%)	23 (25.8%)	9 (30.0%)	6 (30.0%)	0.72
Sheath size > 6 Fr, *n* (%)	43 (46.7%)	36 (40.4%)	13 (43.3%)	9 (45.0%)	0.66
Access vessel diameter (mm), mean ± SD	6.6 ± 1.2	6.8 ± 1.1	6.7 ± 1.0	6.7 ± 1.2	0.41
Calcification at access site, *n* (%)	34 (37.0%)	29 (32.6%)	11 (36.7%)	8 (40.0%)	0.62
Chronic total occlusion, *n* (%)	30 (32.6%)	28 (31.5%)	9 (30.0%)	6 (30.0%)	0.95
In-stent restenosis, *n* (%)	17 (18.5%)	15 (16.9%)	6 (20.0%)	4 (20.0%)	0.87
Lesion length (mm), median [IQR]	52 [38–72]	50 [36–68]	55 [42–70]	58 [40–75]	0.53

Continuous variables are reported as mean ± standard deviation (SD) or median [interquartile range, IQR], and categorical variables as number (%). Statistical comparisons were performed using ANOVA or Kruskal–Wallis tests for continuous variables, and chi-square or Fisher’s exact tests for categorical variables. BMI, body mass index; Fr, French; IQR, interquartile range; SD, standard deviation; VCD, vascular closure device.

**Table 2:** Early Access-Site Complications and Device Failure by Closure Strategy.

Closure Method	Any Complication, *n* (%)	Hematoma, *n* (%)	Pseudoaneurysm, *n* (%)	Thrombosis/Occlusion, *n* (%)	Device Failure, *n* (%)
Manual Compression (*n* = 92)	17 (18.5%)	7 (7.6%)	5 (5.4%)	3 (3.3%)	-
Collagen Plug VCD (*n* = 89)	12 (13.5%)	4 (4.5%)	3 (3.4%)	2 (2.2%)	0 (0.0%)
Suture-Mediated VCD (*n* = 30)	2 (6.7%)	1 (3.3%)	1 (3.3%)	0 (0.0%)	3 (10.0%)
Clip-Based VCD (*n* = 20)	1 (5.0%)	1 (5.0%)	0 (0.0%)	0 (0.0%)	2 (10.0%)

Early access-site complications and device failure rates stratified by closure method in patients with femoral access. Complications include hematoma, pseudoaneurysm, and thrombosis/occlusion occurring within 30 days of the procedure. Device failure was defined as unsuccessful hemostasis requiring additional intervention. Percentages are based on the number of patients in each closure group. VCD, Vascular closure device.

**Table 3:** Predictors of Early Access-Site Complications (≤30 Days)—Multivariable Logistic Regression.

Variable	Adjusted OR	95% CI	*p*-Value
Manual compression (vs. VCD)	2.41	1.06–5.47	0.035
Sheath size > 6 Fr	2.12	1.01–4.47	0.048
Calcification at puncture site	2.74	1.29–5.81	0.009
Access vessel diameter (per mm)	0.89	0.71–1.12	0.33
Chronic total occlusion	1.08	0.52–2.23	0.85
In-stent restenosis	1.15	0.54–2.46	0.72

Multivariable logistic regression identifying independent predictors of early access-site complications occurring within 30 days post procedure. OR are adjusted for baseline anatomical and procedural covariates. A *p*-value < 0.05 was considered statistically significant. CI, confidence interval; OR, odds ratio; VCD, vascular closure device.

## Data Availability

Data available upon request to the corresponding author.

## Abbreviations

The following abbreviations are used in this manuscript:

ASC	Access-site complication
AVF	Arteriovenous fistula
CI	Confidence interval
CT	Computed tomography
CTA	Computed tomography angiography
Fr	French (sheath diameter unit)
ISR	In-stent restenosis
MC	Manual compression
OR	Odds ratio
PAD	Peripheral arterial disease
SD	Standard deviation
VCD	Vascular closure device

## References

[1] Biancari F., D’Andrea V., Di Marco C., Savino G., Tiozzo V., Catania A. (2010). Meta-analysis of randomized trials on the efficacy of vascular closure devices after diagnostic angiography and angioplasty. Am. Heart J..

[2] Nikolsky E., Mehran R., Halkin A., Aymong E.D., Mintz G.S., Lasic Z., Negoita M., Fahy M., Krieger S., Moussa I. (2004). Vascular complications associated with arteriotomy closure devices in patients undergoing percutaneous coronary procedures: A meta-analysis. J. Am. Coll. Cardiol..

[3] Ortiz D., Jahangir A., Singh M., Allaqaband S., Bajwa T.K., Mewissen M.W. (2014). Access site complications after peripheral vascular interventions: Incidence, predictors, and outcomes. Circ. Cardiovasc. Interv..

[4] Cheng T.W., Farber A., King E.G., Levin S.R., Arinze N., Malas M.B., Eslami M.H., Garg K., Rybin D., Siracuse J.J. (2022). Access site complications are uncommon with vascular closure devices or manual compression after lower extremity revascularization. J. Vasc. Surg..

[5] Cacuci A.C., Krankenberg H., Ingwersen M., Gayed M., Stein S.D., Kretzschmar D., Schulze P.C., Thieme M. (2021). Access Site Complications of Peripheral Endovascular Procedures: A Large, Prospective Registry on Predictors and Consequences. J. Endovasc. Ther..

[6] Kennedy S.A., Rajan D.K., Bassett P., Tan K.T., Jaberi A., Mafeld S. (2021). Complication rates associated with antegrade use of vascular closure devices: A systematic review and pooled analysis. J. Vasc. Surg..

[7] Schillinger M., Sabeti S., Loewe C., Dick P., Amighi J., Mlekusch W., Schlager O., Cejna M., Lammer J., Minar E. (2006). Balloon angioplasty versus implantation of nitinol stents in the superficial femoral artery. N. Engl. J. Med..

[8] Noori V.J., Eldrup-Jørgensen J. (2018). A systematic review of vascular closure devices for femoral artery puncture sites. J. Vasc. Surg..

[9] Seto A.H., Abu-Fadel M.S., Sparling J.M., Zacharias S.J., Daly T.S., Harrison A.T., Suh W.M., Vera J.A., Aston C.E., Winters R.J. (2010). Real-time ultrasound guidance facilitates femoral arterial access and reduces vascular complications: FAUST (Femoral Arterial Access With Ultrasound Trial). JACC Cardiovasc. Interv..

[10] Kalish J., Eslami M., Gillespie D., Schermerhorn M., Rybin D., Doros G., Farber A., Vascular Study Group of New England (2015). Routine use of ultrasound guidance in femoral arterial access for peripheral vascular intervention decreases groin hematoma rates. J. Vasc. Surg..

[11] Jolly S.S., AlRashidi S., d’Entremont M.A., Alansari O., Brochu B., Heenan L., Skuriat E., Tyrwhitt J., Raco M., Tsang M. (2022). Routine Ultrasonography Guidance for Femoral Vascular Access for Cardiac Procedures: The UNIVERSAL Randomized Clinical Trial. JAMA Cardiol..

